# Age and fecal microbial strain-specific differences in patients with spondyloarthritis

**DOI:** 10.1186/s13075-018-1510-6

**Published:** 2018-01-30

**Authors:** Matthew L. Stoll, Pamela F. Weiss, Jennifer E. Weiss, Peter A. Nigrovic, Barbara S. Edelheit, S. Lou Bridges, Maria I. Danila, Charles H. Spencer, Marilynn G. Punaro, Kenneth Schikler, Andreas Reiff, Ranjit Kumar, Randy Q. Cron, Casey D. Morrow, Elliot J. Lefkowitz

**Affiliations:** 1 University of Alabama at Birmingham, Birmingham, AL USA; 2 Children’s Hospital of Philadelphia, Philadelphia, PA USA; 3 Hackensack University Medical Center, Hackensack, NJ USA; 4 Boston Children’s Hospital and Brigham and Women’s Hospital, Boston, MA USA; 5 Connecticut Children’s Medical Center, Hartford, CT USA; 6 Nationwide Children’s Hospital, Columbus, OH USA; 7 Texas Scottish Rite Hospital, Dallas, TX USA; 8 University of Louisville, Louisville, KY USA; 9 Children’s Hospital of Los Angeles, Los Angeles, CA USA

**Keywords:** Microbiota, Spondyloarthritis, Sequencing, Bacteroides, Faecalibacterium

## Abstract

**Background:**

Prior studies have demonstrated abnormalities in the composition of the gastrointestinal microbiota in pediatric and adult patients with spondyloarthritis (SpA). In particular, diminished fecal abundance of *Faecalibacterium prausnitzii* and abnormalities in both directions in the abundance of the *Bacteroides* genus have been identified.

**Methods:**

We obtained fecal specimens from 30 children with treatment-naïve enthesitis-related arthritis (ERA) and 19 healthy controls, as well as specimens from 11 adult patients with longstanding SpA and 10 adult healthy controls. All of the samples underwent sequencing of the 16S ribosomal DNA. A subset of the pediatric fecal samples was subjected to shotgun metagenomics sequencing.

**Results:**

ERA patients had decreased abundance of the anti-inflammatory *F. prausnitzii* A2-165 strain (41 ± 28% versus 54 ± 20% of all sequences matching *F. prausnitzii*, *p* = 0.084) and an increased abundance of the control *F. prausnitzii* L2/6 strain (28 ± 28% versus 15 ± 15%, *p* = 0.038). Similar trends were observed in adults with longstanding SpA (n = 11) and controls (*n* = 10). In contrast, the fecal abundance of *Bacteroides fragilis* was increased in ERA subjects (2.0 ± 4.0% versus 0.45 ± 0.7% of all sequences, *p* = 0.045), yet was diminished in adult subjects (0.2 ± % versus 1.0 ± % of all sequences, *p* = 0.106). Shotgun metagenomics sequencing of the fecal DNA in the pediatric subjects revealed diminished coverage of the butanoate pathway (abundance normalized to controls of 1 ± 0.48 versus 0.72 ± 0.33 in ERA, *p* = 0.037).

**Conclusions:**

The anti-inflammatory *F. prausnitzii* A2-165 strain appears to be depleted in both pediatric and adult SpA. In contrast, *B. fragilis* may be depleted in adult disease yet abundant in pediatric SpA, suggesting developmental effects on the immune system.

**Electronic supplementary material:**

The online version of this article (10.1186/s13075-018-1510-6) contains supplementary material, which is available to authorized users.

## Background

The role of the intestinal microbiota in the pathogenesis of spondyloarthritis (SpA) is gaining widespread interest [1]. Much of this interest was spurred by a large body of literature indicating abnormalities of the microbiota in patients with inflammatory bowel disease (IBD) [2], a condition in which therapeutic alteration of the microbiota in the form of antibiotics, probiotics, and even fecal transplant may be effective [3], along with the clinical and genetic associations between IBD and SpA [4]. Prior studies evaluating fecal or mucosal microbiota of adult patients with SpA demonstrated diminished fecal abundance of *Bacteroides* [5, 6], with one study showing a potential association between the *Dialister* genus and disease severity [6]. There are comparatively few data reported in pediatric SpA. One study showed decreased abundance of *Prevotella* in pediatric SpA [7], and our prior work showed decreased abundance of *Faecalibacterium prausnitzii* [8]. The latter finding is consistent with multiple similar reports in subjects with IBD [9]. Depletion of *F. prausnitzii* may adversely impact intestinal health through diminished production of butyrate and other short chain fatty acids [10], and our previous work has shown diminished abundance of butyrate in the fecal water of children with ERA [11]. Both pediatric SpA studies also showed increased abundance of unspecified species within the *Bacteroides* genus, consistent with studies in children with other categories of juvenile idiopathic arthritis (JIA) [12, 13], yet the opposite of findings in adult subjects with rheumatoid arthritis (RA) [14, 15] or SpA [5, 6].

Herein, we studied the intestinal microbiota in children with treatment-naïve SpA in comparison to pediatric healthy controls; to do this, we used a novel bioinformatics tool in combination with the Basic Local Alignment Search Tool (BLAST) that permitted assessment of species and even strain-level identification of certain organisms, as different strains within a species can have dramatically different effects [16, 17]. In order to validate our prior results and to further increase the sample size, we recruited subjects from geographic locations around the country. Prior studies have shown that geography can impact the microbiota in situations in which subjects with highly different environments (e.g., urban versus rural) and ethnicities are compared [18, 19], but there are no data evaluating subjects from different urban areas specifically within the United States. The findings in these subjects were compared with those in adults with longstanding SpA and adult controls, to identify which changes might be common to SpA and which might be unique to pediatric populations. We also performed shotgun metagenomics sequencing on a subset of children with juvenile SpA in order to obtain additional information regarding potential mechanisms whereby the microbiota might predispose to SpA. Herein, we demonstrate that the anti-inflammatory A2-165 strain of *F. prausnitzii* is depleted in both pediatric and adult SpA, and that the microbiota of children with SpA has decreased genetic capacity to synthesize butyrate. We additionally demonstrate that *B. fragilis* is depleted in adult SpA yet abundant in pediatric disease.

## Methods

### Subjects

Pediatric SpA subjects were mostly children with enthesitis-related arthritis (ERA; juvenile SpA) as per the International League of Association for Rheumatology criteria [20]; three with sacroiliitis in the absence of peripheral arthritis met the Assessment of Spondyloarthritis International Society (ASAS) criteria for axial SpA [21]. Pediatric controls were either healthy children recruited through advertisements or children referred to rheumatology for evaluation of arthritis but found to have noninflammatory causes of joint pain or irrelevant laboratory markers, such as a positive antinuclear antibody. None of the controls had any findings suggestive of infectious arthropathies, including Lyme synovitis. Pediatric subjects were recruited from eight sites around the country (Table 1). Exclusion criteria were receipt of antibiotics within 3 months prior to study enrollment and prior treatment with any immunosuppressive agent excluding less than 2 weeks of corticosteroids. None of the pediatric subjects or controls had been included in our prior work [8].

**Table 1:** Demographic and clinical characteristics of pediatric study participants

Characteristic	ERA	Controls
*n*	30	19
Age (years), mean ± SD	13.5 ± 3.0	13.6 ± 2.7
BMI (kg/m^2^), mean ± SD	20.7 ± 4.1	21.5 ± 6.1
Male	19 (63%)	13 (68%)
Race		
Caucasian	23 (77%)	17 (89%)
African-American	3 (10%)	2 (10%)
Asian	2 (6.7%)	0
Other/unknown	2 (6.7%)	0
Site		
UAB (Birmingham, AL, USA)	8 (27%)	9 (47%)
CHOP (Philadelphia, PA, USA)	10 (33%)	4 (21%)
HUMC (Hackensack, NJ, USA)	1 (3.3%)	4 (21%)
CCMC (Hartford, CT, USA)	1 (3.3%)	2 (10%)
BCH (Boston, MA, USA)	4 (13%)	0
NCH (Columbus, OH, USA)	2 (6.7%)	0
TSRH (Dallas, TX, USA)	2 (6.7%)	0
CHLA (Los Angeles, CA, USA)	1 (3.3%)	0
UL (Louisville, KY, USA)	1 (3.3%)	0
HLA-B27^+^	15/29 (52%)	Not measured
Sacroiliitis	18 (60%)	None
Clinical only	8/18 (44%)	
Imaging	10/18 (56%)	

Data presented as *n* (%) unless stated otherwise

*BCH* Boston Children’s Hospital, *BMI* body mass index, *CHLA* Children’s Hospital of Los Angeles, CCMC Connecticut Children’s Medical Center, *CHOP* Children’s Hospital of Philadelphia, *ERA* enthesitis-related arthritis, *HUMC* Hackensack University Medical Center, *NCH* Nationwide Children’s Hospital, *TSRH* Texas Scottish Rite Hospital, *UAB* University of Alabama at Birmingham, *UL* University of Louisville

Adult SpA subjects were all recruited at the University of Alabama at Birmingham (UAB). Patients met either the ASAS criteria for peripheral [22] or axial [21] SpA or the Classification Criteria for Psoriatic Arthritis (CASPAR) criteria [23] for psoriatic arthritis (PsA). Sacroiliitis in both children and adults was defined based upon either clinical findings per the judgment of the treating rheumatologist or imaging findings on MRI or radiography; only a single observer was required. Adult controls were healthy volunteers recruited through advertisements or patients with noninflammatory causes of joint pain (Table 2). For the adult subjects, as with the pediatric counterparts, recent antibiotic use was an exclusion criterion; however, for the adult subjects, immunosuppressive therapy was not.

**Table 2:** Demographic and clinical characteristic of adult study participants

Characteristic	SpA	Controls
*n*	11	10
Age (years), mean ± SD	52 ± 7.4	47 ± 8.1
BMI (kg/m^2^), mean ± SD	32 ± 7.8	28 ± 4.8
Disease duration (years), mean ± SD	11 ± 9.5	Not applicable
Male	4 (36%)	3 (30%)
Race		
Caucasian	9 (82%)	6 (60%)
African-American	2 (18%)	4 (40%)
Diagnosis		Not applicable
Undifferentiated SpA	1 (9.1%)	
Ankylosing spondylitis	6 (54%)	
IBD-associated arthritis	1 (9.1%)	
Psoriatic arthritis	2 (18%)	
Reactive arthritis	1 (9.1%)	
Current medications		
None	2 (18%)	10 (100%)
Traditional DMARDs alone	5 (45%)	
TNFi alone	3 (27%)	
DMARDs plus TNFi	1 (9.1%)	
HLA-B27^+^	3/3 (100%)	Not measured
Sacroiliitis	8 (73%)	None
Clinical	1 (12%)	
Imaging	7 (88%)	

Data presented as *n* (%) unless stated otherwise

*BMI* body mass index, *DMARD* disease-modifying anti-rheumatic drug (leflunomide, *n* = 2; methotrexate, *n* = 2; sulfasalazine, *n* = 3), *IBD* inflammatory bowel disease, *SpA* spondyloarthritis, *TNFi* tumor necrosis factor inhibitor

### Processing of fecal samples

Subjects collected the samples at home and immediately placed the samples in a 50-ml container filled with Cary-Blair media. Samples were shipped overnight via commercial carrier to the microbiota core at UAB. Microbial genomic DNA was isolated by standard methods using kits from Zymo Research (Irvine, CA, USA) as per the manufacturer’s instructions. For 16S sequencing, the Zymo ZR Fecal DNA MiniPrep kit (catalog # D6010) was used; for shotgun metagenomics, the whole genome DNA kit (catalog # D6110) was used.

### Sequencing and analysis of 16S ribosomal DNA from the fecal specimens

The purified DNA (~100 ng) underwent PCR amplification using primers designed to the conserved region flanking the V4 region from the 16S ribosomal DNA (rDNA) gene, as described previously [8]. Resulting PCR fragments were run on the Illumina MiSeq (San Diego, CA, USA) at a concentration of 12 pM; read lengths were approximately 250-bp paired-end reads. Initial steps of the analysis were performed with the Divisive Amplicon Denoising Algorithm (DADA2) [24, 25]. Unlike some of the widely used algorithms [26, 27], DADA2 does not cluster similar sequences. Instead, it uses error modeling to distinguish amplification or sequencing errors from true sequences, which are typically referred to as sequence variants (SVs). This assessment is based upon features of the sequence, specifically the distance from its nearest neighbor as measured by the number of nucleotide differences and as influenced by quality scores and specific nucleotide transitions; and its abundance. Thus, a highly abundant sequence may be assessed as a true SV rather than a sequence error, even if it is highly similar to another abundant SV. Consequently, DADA2 can identify unique strains within a species. The output of DADA2 is an abundance table, in which each unique sequence is characterized by its abundance in each sample. Taxonomic information was incorporated into this abundance table with the ribosomal database project naïve Bayesian classifier [28] using the May 2013 version of Greengenes [29]. This abundance table has the same structure as a traditional operational taxonomic unit table that is the analytic unit in the Quantitative Insight Into Microbial Ecology (QIIME) tool suite [26]. Thus, it was incorporated into QIIME with the biom convert script, and assessment of alpha and beta diversity was performed within QIIME. Alpha diversity indexes of richness and evenness were assessed with the Chao1 and Shannon measures respectively; beta diversity was assessed with the Bray Curtis measure, visualized using principal coordinates analysis (PCoA). Variables assessed were presence versus absence of arthritis, and among the ERA patients were geographic location, presence versus absence of sacroiliitis, and HLA-B27 status. Geographic location was assessed both by looking at each site as a separate unit and by pooling into one region the four sites located in the northeast (Boston Children’s Hospital, Connecticut Children’s Medical Center, Hackensack University Medical Center, and Children’s Hospital of Philadelphia), a geographic area that is similar in size to the catchment area of UAB. To identify specific strains within *F. prausnitzii*, the 16S sequences were downloaded from the National Library of Medicine, and the SVs matching *F. prausnitzii* were aligned against the known sequences using BLAST. The code used for the 16S sequence analysis is available in Additional file 1.

### Sequencing and analysis of shotgun sequencing from the fecal specimens

The purified DNA was sheared and 500–600-bp fragments were purified by agarose gel electrophoresis. Illumina kits were used for adapter ligation followed by Illumina HiSeq, 125-bp paired-end sequencing. Approximately 25–30 million reads per sample were obtained from each sample. A second set was run on the Illumina MiSeq, yielding 3–7 million reads per sample, approximately 250-bp paired-end sequencing. Quality control of both sets was performed using PRINSEQ [30]. Alignment with the human genome to remove human reads was performed with Bowtie2 [31] using the very-sensitive mode. Conversion of fastq to fasta files was performed with the FASTX tool kit (http://hannonlab.cshl.edu/fastx_toolkit/commandline.html). The remainder of the analysis was performed with the HMP Unified Metabolic Analysis Network (HUMAnN V2) program [32]. A detailed description of the program is available online (http://huttenhower.sph.harvard.edu/humann2). Briefly, the input fasta files were aligned against a functionally annotated pan-genome database of over 4000 species, named ChocoPhlan by the HUMAnN2 investigators, using Bowtie2 [31]. Reads not mapped to ChocoPhlan were subsequently aligned against the UniProt universal proteins database [33] using double index alignment of next-generation sequencing data (DIAMOND) [34], a more efficient alternative to BLASTX. Pathway assignments were performed with MetaCyc [35]; all genes associated with a pathway must be present for the pathway to be considered a match. Gene and pathway abundances were normalized to the sequencing depth prior to any further analyses. Translation from UniProt to Kyoto Encyclopedia of Genes and Genomes (KEGG) [36] was performed with the map_ko_uniref50.txt.gz mapping file made available by the HUMAnN investigators. The output of HUMAnN2 is files containing the abundance of each gene as well as of each pathway, optionally normalized to the sequencing depth of each subject. The code used for the HUMAnN2 analysis is included in Additional file 1.

### Statistical analysis

To evaluate whether the samples as a whole clustered according to nominal variables, the permutation multivariate analysis of variance (PERMANOVA) test was run against the distance matrix generated from the Bray Curtis test of beta diversity. The PERMANOVA test partitions a distance matrix among sources of variation (e.g., presence versus absence of arthritis) in order to describe the strength and significance that a categorical variable has in determining the variation of distances [37]. Comparisons in the abundance of specific taxa, as well as assessments of differences in alpha diversity, were performed with Student’s *t* test. Pairwise comparisons performed at each of the phylogenetic levels (e.g., phylum) were corrected for multiple comparisons with the Benjamini–Hochberg false discovery rate (FDR) test [38] with a significance threshold of 0.05.

## Results

### Subjects

The demographic and clinical features of the pediatric subjects are summarized in Table 1. All were naïve to immunosuppressive therapy, and none had been exposed to systemic antibiotics within 3 months prior to enrollment in the study. The patients and controls were well matched for age and BMI.

### 16S sequencing

A mean of 129,226 (range 39,741–249,043) paired-end reads were obtained from the 49 subjects. Following quality filtering, merging, and chimera removal, a total of 64,016 (range 23,942–105,497) reads were used. Among the 49 subjects, a total of 1402 unique sequences (sequence variants (SVs)) were obtained; as most of these were rare and unique to specific subjects, the range was 46–236 SVs/subject.

**Fig. 1:**
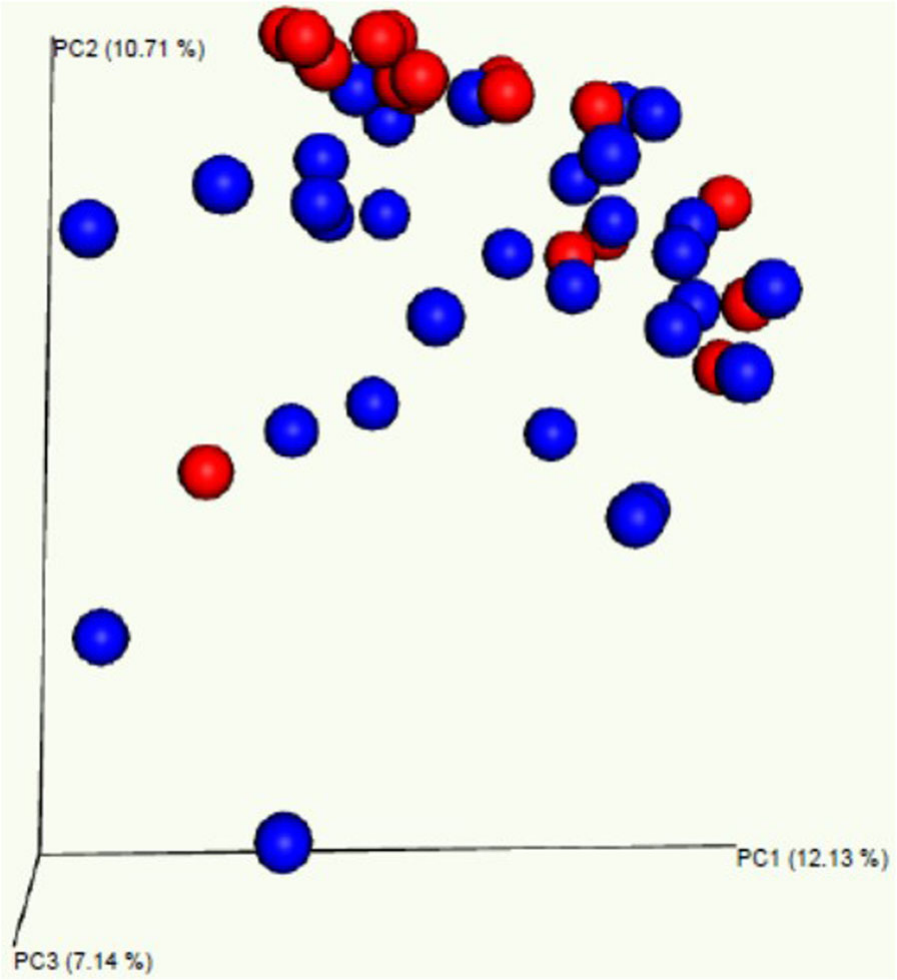
Principal coordinates analysis of the 16S sequencing results obtained from children with ERA and pediatric healthy controls demonstrating clustering by diagnosis. Blue and red dots reflect ERA patients and controls, respectively

PCoA on the 49 subjects demonstrated clustering by diagnosis (ERA versus control; Fig. 1). This visually evident clustering was confirmed by the PERMANOVA test (*F* = 1.45, *p* = 0.046). Among the ERA patients, no sources of variation were observed on the basis of any single variable, including geographic location (Bray Curtis *F* = 1.09, *p* = 0.277), HLA-B27 status (Bray Curtis *F* = 0.902, *p* = 0.652), or the presence of sacroiliitis (Bray Curtis *F* = 0.471, *p* = 1). Geographic location was assessed both by looking at each site as a separate unit and by pooling into one region the four sites located in the northeast (Boston Children’s Hospital, Connecticut Children’s Medical Center, Hackensack University Medical Center, and Children’s Hospital of Philadelphia), a geographic area that is similar in size to the catchment area of UAB.

Due to the large number of SVs as already indicated, pairwise comparisons at most of the phylogenetic levels failed to identify any groupwise differences following adjustment for multiple comparisons. The only exception was that ERA patients demonstrated decreased abundance of the Actinobacteria phylum as compared to controls (3.4% versus 9.3%, *p* = 0.002 uncorrected, *p* = 0.05 FDR-corrected). This lack of any significant pairwise differences held even after SVs within a species were collapsed into the species, thus reducing the number of unique items to 366. Consequently, based upon previous work in children with ERA [7, 8], the analysis was focused on two organisms: *F. prausnitzii* and the *Bacteroides* genus, particularly *B. fragilis*.

**Fig. 2:**
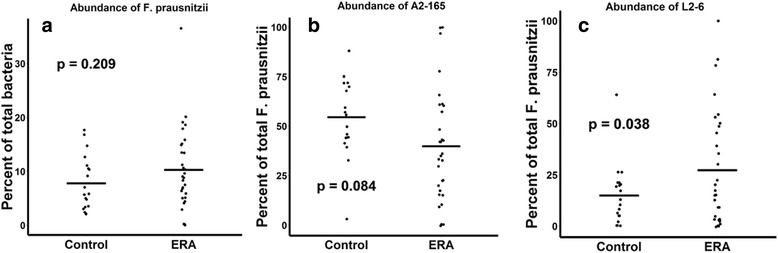
Strain-level variation in the fecal abundance of *Faecalibacterium prausnitzii* in children with ERA and pediatric controls. Abundance of *F. prausnitzii* as a percentage of total fecal content is similar in patients and controls (**a**). As a percentage of total *F. prausnitzii*, children with ERA have a trend toward decreased fecal abundance of the anti-inflammatory A2-165 strain (**b**) as well as increased abundance of the L2/6 strain (**c**). ERA enthesitis-related arthritis

The abundance of *F. prausnitzii* as a whole was nominally higher in the patients (10 versus 7.8%, *p* = 0.192; Fig. 2a), in contrast to our previous results [8]. Twenty-three unique SVs were identified within *F. prausnitzii*. As discussed in Methods, BLAST was used to identify strains within the *F. prausnitzii* SVs identified by DADA2. Of those 23 SVs, one of them was a 100% best match (253/253 bp) with strain A2-165, which has been shown previously to have regulatory effects [39]. Recent studies have demonstrated that even compared to other strains of *F. prausnitzii*, such as L2/6, the A2-165 strain induces increased interleukin-10 production from peripheral blood mononuclear cells [16, 40] and has increased butyrate promoter activity [41]. A separate SV was a 99% best match (252/253 bp) with L2/6, the comparator strain in some of the aforementioned studies. These two strains were the two most abundant SVs matching *F. prausnitzii*. As a proportion of the sequences that matched *F. prausnitzii*, ERA patients had relatively decreased abundance of the regulatory A2-165 strain (41 ± 28% versus 54 ± 20%, *p* = 0.084; Fig. 2b) and a relatively increased abundance of the SV that most closely matched L2/6 (28 ± 28% versus 15 ± 15%, *p* = 0.038; Fic 2c). Thus, the stool samples from pediatric ERA patients contained a decreased ratio of the antiinflammatory, relative to the neutral, strain of *F. prausnitzii*.

**Fig. 3:**
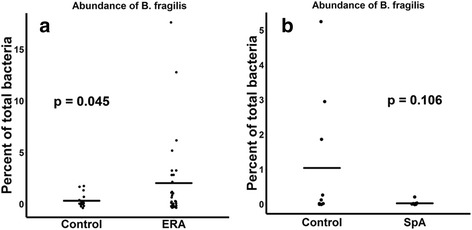
Fecal abundance of *Bacteroides fragilis* in pediatric and adult subjects with SpA. Increased fecal abundance is observed in ERA patients compared to pediatric controls (**a**), while a trend toward decreased fecal abundance was observed in adult SpA patients compared to adult controls (**b**). ERA enthesitis-related arthritis, SpA spondyloarthritis

ERA patients and controls had similar levels of the *Bacteroides* genus (20 versus 19%). However, among the *Bacteroides* genus, *B. fragilis* is considered to have regulatory properties through its polysaccharide tail [42] and thus was investigated further. Here, consistent with our previous findings [8], ERA patients had a four-fold increased abundance (2.0 ± 4.0% versus 0.45 0.7%, *p* = 0.045; Fig. 3a). There were no significant differences with any of the other species of *Bacteroides*. In summary, children with ERA had diminished abundance of the regulatory A2-165 strain of *F. prausnitzii* as well as increased abundance of *B. fragilis*, compared to healthy control subjects.

To evaluate whether these strain-level and species-level differences between arthritis subjects and controls are unique to pediatric subjects or are potentially a marker of SpA as a whole, 16S sequencing was run on a cohort of 11 adults with longstanding SpA and 10 healthy adult controls. Characteristics of the adult subjects are presented in Table 2; most had longstanding disease, with a mean disease duration of 11 years, and most were on immunosuppressive therapy. As with the pediatric subjects, none of the adult SpA patient or controls had been exposed to systemic antibiotics within 3 months of enrollment.

Among the adults, the opposite trend with respect to *Bacteroides* abundance was seen. Specifically, *Bacteroides* abundance as a proportion of total sequencing depth was 11 ± 9% among the patients, compared to 26 ± 18% in the controls (*p* = 0.036), although there were no significant differences in the abundance of *B. fragilis* (0.2 ± 0.6% in patients versus 1 ± 1.8% in controls, *p* = 0.106) (Fig. 3b). In contrast, the trends involving *F. prausnitzii* strains were similar in adults as compared to children (total *F. prausnitzii* 6.9 ± 6.9% in controls versus 10 ± 10% in patients (*p* = 0.427); A2-165 as percentage of *F. prausnitzii* 41 ± 30% in controls versus 25 ± 13% in patients (*p* = 0.175)), although the differences were not statistically significant, possibly due to a smaller sample size. Similar trends for both organisms were observed if the three subjects taking sulfasalazine were excluded (data not shown.)

Finally, the abundance of additional organisms that have emerged as being of interest in SpA was evaluated. Specifically, the Lachnospiraceae family was reduced in children with ERA in our previous work [8], yet was elevated in both biopsy [43] and fecal [44] specimens in prior studies of adults with SpA. There were, however, no differences in the abundance of this family in either the pediatric or the adult subjects (data not shown). A study of ileal biopsies demonstrated that the abundance of the *Dialister* genus correlated with disease activity in treatment-naïve SpA patients [6]; this organism was virtually undetectable (<1%) in the fecal specimens of all subjects in both age groups, likely indicating specificity to mucosal specimens. *Ruminococcus gnavus* was elevated in the feces of adult subjects with SpA in a prior report [44], but the differences of this organism herein were not statistically significant in either age group. Finally, both Stebbings et al. [5] and our group [8] have shown increased abundance of *Bifidobacterium* in the feces of SpA patients; surprisingly, the same genus was depleted among pediatric ERA subjects compared to healthy controls (2.6% versus 7.2%, *p* = 0.008), with no differences among the adult subjects.

### Whole genome sequencing

**Fig. 4:**
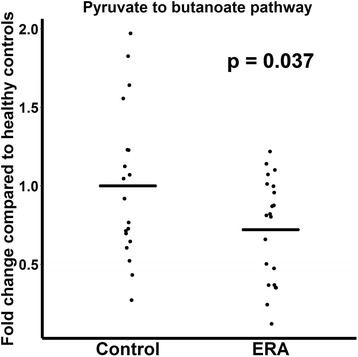
Fecal abundance of bacterial genes comprising a pathway involved in butyrate synthesis. Results of two separate studies shown. For each, each subject was normalized to the mean of the healthy controls for that study, and the combined results are shown. ERA enthesitis-related arthritis

To assess the potential functional consequences of the presented findings, whole genome sequencing of fecal DNA with the HiSeq device was performed on a subset of the aforementioned pediatric patients, consisting of 14 patients each (ERA and healthy controls), supplemented by 12 additional subjects (five controls and seven ERA patients) who were sequenced with the MiSeq device. These subjects were similar with respect to demographic characteristics and BMI (Table 3). A mean of 23 million (range 14.7–38.7 million) high-quality sequences from the Illumina HiSeq sequencer was analyzed, as was a mean of 5.3 million (range 3.5–7.8 million) high-quality sequences from the MiSeq device. There was no obvious clustering based upon diagnosis at either the level of individual genes or whole pathways (data not shown), consistent with a core microbiome not affected by the disease state [45]. In light of our findings in the present study and our previous report [8] showing alterations in the fecal abundance of *F. prausnitzii*, we specifically examined the butanoate pathway (MetaCyc title CENTFERM-PWY pyruvate fermentation to butanoate), which results in synthesis of butyrate from precursors such as pyruvate and acetyl-CoA. With both devices, there was a modest difference in the abundance of this pathway. For both the MiSeq and HiSeq devices, each value was normalized to the mean value for the controls for the respective run, and the two sets were pooled, revealing a statistically significant decreased representation of the butanoate pathway in ERA patients compared to controls (1 ± 0.48 versus 0.72 ± 0.33, *p* = 0.037; Fig. 4.) Thus, our data indicate that the fecal microbiota of ERA patients compared to controls had decreased capacity to synthesize a compound that is generally considered to have anti-inflammatory properties within the intestinal environment [46].

**Table 3:** Demographic and clinical characteristics of participants undergoing whole genome shotgun sequencing

Characteristic	ERA	Controls
*n*	21	19
Age (years), mean ± SD	13.6 ± 3.0	12.9 ± 3.0
BMI (kg/m^2^), mean ± SD	21.0 ± 5.1	20.4 ± 6.2
Male	12 (57%)	12 (63%)
Race		
Caucasian	14 (67%)	14 (74%)
African-American	5 (24%)	5 (26%)
Asian	2 (9.5%)	0
Site		
UAB (Birmingham, AL, USA)	8 (38%)	11 (58%)
CHOP (Philadelphia, PA, USA)	7 (33%)	2 (10%)
HUMC (Hackensack, NJ, USA)	1 (4.8%)	2 (10%)
CCMC (Hartford, CT, USA)	1 (4.8%)	4 (21%)
BCH (Boston, MA, USA)	4 (19%)	0
HLA-B27^+^	10/20 (50%)	Not measured
Sacroiliitis	10 (48%)	None
Clinical only	4 (40%)	
Imaging	6 (60%)	

Data presented as *n* (%) unless stated otherwise

*BCH* Boston Children’s Hospital, *BMI* body mass index, *CCMC* Connecticut Children’s Medical Center, *CHOP* Children’s Hospital of Philadelphia, *ERA* enthesitis-related arthritis, *HUMC* Hackensack University Medical Center, *UAB* University of Alabama at Birmingham

## Discussion

Mechanisms by which an altered microbiota might be associated with inflammatory arthritis are as yet unknown. Animal studies clearly demonstrate a causal relationship, as an animal model of SpA is abrogated in the germ-free environment [47]. Herein, we report strain-level differences in fecal abundance of *F. prausnitzii* between children with ERA and healthy controls. Additionally, at the functional level, decreased genetic abundance of the butanoate pathway was observed in children with ERA. Children with ERA also had increased abundance of *B. fragilis*. Notably, adults with longstanding SpA had similar trends with respect to *F. prausnitzii* abundance, but opposite trends with respect to *Bacteroides*, with decreased abundance of *Bacteroides* genus seen in adult SpA patients compared to adult healthy controls.

This work supports a potential role of *F. prausnitzii* in the pathogenesis of SpA. Although previous studies assessing the microbiota in adults with SpA have not reported alterations in the abundance of this organism [5, 6, 43], this was reported previously in a pediatric study of SpA patients [8]. This particular species is generally considered to have anti-inflammatory effects through production of SCFAs, such as butyrate [10], or by direct effects on cytokine production [39], and our results demonstrated decreased genetic capacity to synthesize butyrate among ERA patients compared to healthy control pediatric subjects. SCFAs serve as major sources of energy for the intestinal enterocytes and also regulate the differentiation of T cells, promoting a regulatory phenotype [46, 48]. A meta-analysis of studies of patients with IBD showed decreased intestinal abundance of *F. prausnitzii* [9]. Our work extends these previous observations, by showing that the specific strain within *F. prausnitzii* may be of particular relevance. Recent studies indicated that the A2-165 strain, as compared to L2/6 and other strains, may have greater capacity to induce IL-10 production [40] and has increased promoter activity at the butyryl-CoA CoA transferase gene, which codes for the enzyme that catalyzes the final step in the butyrate synthesis pathway [41]. Thus, not only did ERA patients demonstrate less representation of the butanoate pathway, but their promoter activity at a key locus determining butyrate production may also be diminished. Not surprisingly in light of these results, a study of fecal metabolomics demonstrated diminished representation of the butanoate pathway in ERA patients compared to controls [11].

Another important finding in this work pertained to the abundance of *Bacteroides*. Consistent with previous observations in children with multiple forms of JIA including ERA [7, 8, 13], this organism was relatively overrepresented among pediatric SpA patients. This finding appears to be uniquely associated with pediatric subjects; several studies in adults with both RA [14, 15, 49] and SpA [5, 6] have shown decreased abundance of *Bacteroides* in patients compared to healthy controls. Similar to these other reports, adult SpA patients in this study showed decreased abundance of *Bacteroides*.

One potential way to reconcile these findings is that *B. fragilis* may contribute to JIA not by directly causing an inflammatory process, but rather through its effects on the ontogeny of the immune system. Vatanen et al. [50] compared the fecal microbiota of children up to age 3 years in areas with high (Estonia and Finland) and low (Russia) incidences of pediatric autoimmunity, finding among the differences decreased fecal abundance of *Bacteroides*, particularly *Bacteroides dorei*, in the Russian children. That the differences in the incidence of autoimmunity may have been related to the abundance of *Bacteroides* was suggested by findings that the lipopolysaccharide (LPS) of *B. dorei* generated a less robust inflammatory reaction than did that of *Escherichia coli* yet failed to induce tolerance to repeated doses of endotoxin; furthermore, the LPS of *B. dorei*, unlike that of *E. coli*, did not prevent development of type 1 diabetes when injected intraperitoneally into young nonobese diabetic mice. Thus, elevated fecal abundance of *Bacteroides* may represent an illustration of the hygiene hypothesis, which posits that abnormal early life microbial exposures result in altered immune priming and consequent increased risk of autoimmune diseases [51].

The findings of our study need to be interpreted in view of its limitations. Despite including subjects from around the country, the sample size was small, and we cannot exclude geographic contributions to the findings. HLA-B27 itself may also have contributed to the variance [52], although among the ERA patients there was no evident clustering based upon the presence versus absence of this marker, and there is to our knowledge no published data indicating that the HLA-B27 allele affects the microbiota in humans. In addition, all adult participants with longstanding SpA had been exposed to a variety of immunosuppressive therapies. Furthermore, three of them had been exposed to sulfasalazine, which is in part an antibiotic; however, excluding them did not alter our findings. We also do not have information on subclinical intestinal inflammation, which is frequently present in patients with SpA [53]. Finally, many of the adult and pediatric controls had been referred to evaluate for arthritis; however, these subjects were evaluated by an attending pediatric or adult rheumatologist, who excluded a diagnosis of arthritis on the basis of the historical and physical examination findings. The strengths of the study are that the pediatric arm was limited to treatment-naïve subjects, we used a novel informatics approach to identify strain-level differences between patients and controls, and we included shotgun sequencing data that corroborated the 16S findings.

## Conclusion

Our study supports previous work indicating that decreased fecal abundance of a regulatory strain of *F. prausnitzii* may be at least partly responsible for the pathogenesis of SpA, possibly due to decreased production of butyrate, and that efforts to replenish it in patients with SpA may be a potential therapeutic avenue. In contrast, to the extent that increased abundance of *Bacteroides* or *B. fragilis* in children reflects altered immunologic development rather than direct pathogenicity or the organism, enthusiasm for microbial-based interventions to address this organism may be tempered. Instead, our findings may underscore the necessity for prevention efforts, such as avoiding unnecessary use of antibiotics in healthy children [54, 55].

## Additional file

Additional file 1: Code for processing of 16S sequences with DADA2 and for shotgun sequence analysis with HUMAnN2. (DOCX 12 kb)

## Abbreviations

ASAS: Assessment of Spondyloarthritis International Society
BLAST: Basic Local Alignment Search Tool
CASPAR: Classification Criteria for Psoriatic Arthritis
DADA: Divisive Amplicon Denoising Algorithm
DIAMOND: double index alignment of next-generation sequencing data
ERA: Enthesitis-related arthritis
FDR: False discovery rate
HUMAnN: HMP Unified Metabolic Analysis
IBD: Inflammatory bowel disease
JIA: Juvenile idiopathic arthritis
KEGG: Kyoto Encyclopedia of Genes and Genomes
LPS: Lipopolysaccharide
PCoA: Principal coordinates analysis
PERMANOVA: Permutation multivariate analysis of variance
PsA: Psoriatic arthritis
QIIME: Quantitative Insight Into Microbial Ecology
RA: Rheumatoid arthritis
rDNA: Ribosomal DNA
SCFA: Short chain fatty acids
UAB: University of Alabama at Birmingham
