# Supplementary material for: Age and fecal microbial strain-specific differences in patients with spondyloarthritis
Source: Arthritis Res Ther. 2018 Jan 30;20:14. doi: 10.1186/s13075-018-1510-6 (PMC5791354; doi:10.1186/s13075-018-1510-6)
Supplement: Additional file 1: — Code for processing of 16S sequences with DADA2 and for shotgun sequence analysis with HUMAnN2. (DOCX 12 kb) [file 13075_2018_1510_MOESM1_ESM.docx]

Processing of 16S sequences with DADA2

# Upload the packages.

library(dada2); packageVersion("dada2")

library(ShortRead); packageVersion("ShortRead")

library(ggplot2); packageVersion("ggplot2")

# fnFs refers to all the forward files.

# fnR2 refers to all the reverse files.

# The filtering step.

for(i in seq_along(fnFs)) {

fastqPairedFilter(c(fnFs[i], fnRs[i]), c(filtFs[i], filtRs[i]),

truncLen=c(200,140),# Forward and Reverse

maxN=0, maxEE=c(1,1), truncQ=2,

compress=TRUE, verbose=TRUE)

}

# Dereplication

derepFs <- derepFastq(filtFs, verbose=TRUE)

derepRs <- derepFastq(filtRs, verbose=TRUE)

names(derepFs) <- sample.names

names(derepRs) <- sample.names

# This changes the default values for the dada call.

setDadaOpt(OMEGA_A = 1e-50,MAX_CONSIST=25)

# Now the error learning step.

dadaFs.lrn <- dada(derepFs, err=NULL, selfConsist = TRUE, multithread=TRUE)

errF <- dadaFs.lrn[[1]]$err_out

dadaRs.lrn <- dada(derepRs, err=NULL, selfConsist = TRUE, multithread=TRUE)

errR <- dadaRs.lrn[[1]]$err_out

# Merging of sequences; no discrepancies are permitted in the area of overlap.

mergers <- mergePairs(dadaFs, derepFs, dadaRs, derepRs, verbose=TRUE)

# Make a sequence table

seqtab <- makeSequenceTable(mergers[names(mergers) != "Mock"])

# Remove chimeras

seqtab.nochim <- removeBimeraDenovo(seqtab, verbose=TRUE)

# Now assign taxonomy, using the most recent gg database

taxa <- assignTaxonomy(seqtab.nochim, "gg_13_8_train_set_97.fa.gz")

Shotgun metagenomics analysis

humann2 --input $SUBJECT.fasta --output $SUBJECT_outputoutput --thread 12 --protein-database /PATH_TO_HUMANN2/humann2/data/FULL_DATABASE/uniref50_diamond/uniref/ --translated-query-coverage-threshold 40 --translated-subject-coverage-threshold 50.0 --identity-threshold 40.0
